# PiER: web-based facilities tailored for genetic target prioritisation harnessing human disease genetics, functional genomics and protein interactions

**DOI:** 10.1093/nar/gkac379

**Published:** 2022-05-24

**Authors:** Hai Fang

**Affiliations:** Shanghai Institute of Hematology, State Key Laboratory of Medical Genomics, National Research Center for Translational Medicine at Shanghai, Ruijin Hospital, Shanghai Jiao Tong University School of Medicine, Shanghai 200025, China

## Abstract

Integrative prioritisation promotes translational use of disease genetic findings in target discovery. I report ‘PiER’ (http://www.genetictargets.com/PiER), web-based facilities that support *ab initio* and *real-time* genetic target prioritisation through integrative use of human disease genetics, functional genomics and protein interactions. By design, the PiER features two facilities: *elementary* and *combinatory*. The elementary facility is designed to perform specific tasks, including three online tools: *eV2CG*, utilising functional genomics to link disease-associated variants (particularly located at the non-coding genome) to core genes likely responsible for genetic associations in disease; *eCG2PG*, using knowledge of protein interactions to ‘network’ core genes and additional peripheral genes, producing a ranked list of core and peripheral genes; and *eCrosstalk*, exploiting the information of pathway-derived interactions to identify highly-ranked genes mediating crosstalk between molecular pathways. Each of elementary tasks giving results is sequentially piped to the next one. By chaining together elementary tasks, the combinatory facility automates genetics-led and network-based integrative prioritisation for genetic targets at the gene level (*cTGene*) and at the crosstalk level (*cTCrosstalk*). Together with a tutorial-like booklet describing instructions on how to use, the PiER facilities meet multi-tasking needs to accelerate computational translational medicine that leverages human disease genetics and genomics for early-stage target discovery and drug repurposing.

## Graphical Abstract

**Graphical Abstract:**
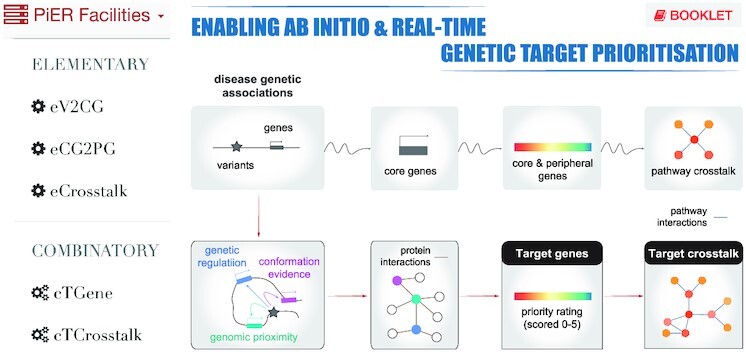
Overview of the online tools provided by the PiER facilities that enable *ab initio* and *real-time* genetic target prioritisation at the gene and crosstalk levels leveraging disease genetics.

## INTRODUCTION

Genetic targets, defined as genetically informed target candidates, are increasingly appreciated for their importance in enhancing late-stage drug approval. Retrospective analyses show that drug-target pairs with human disease genetic evidence are twice as likely to be therapeutically successful as those without such evidence (1–3). The success rate is even higher for drugs supported by genetic targets with causal relation to disease (4).

The field of target discovery has been advanced by genetically driven target prioritisation approaches (5,6). Integrative prioritisation for early-stage genetic target discovery has proven cost-effective in promoting the translational use of disease genetic associations [i.e. summary-level data arising from genome-wide association studies (GWAS) (7–9)], which is increasingly recognised in reducing drug attrition rate in late-stage clinical trials. As a general guidance, effective prioritisation is likely to use integrative predictors that incorporate multi-layered functional genomic data and knowledge of protein interactions as well. Firstly, incorporating functional genomics is motivated by the intrinsic difficulty in linking disease-associated variants (mostly located at the non-coding genome for common disease) to candidate genes. This difficulty can be resolved by measuring regulatory effects of non-coding variants on genes. Such effects are likely to modulate genes via long-range chromatin conformations and to act in a cell-type-specific manner. The effects of variants on gene regulation can be mapped in promoter capture Hi-C (PCHi-C) studies that capture long-range physical interactions with gene promoters (i.e. conformation evidence) (10). These effects can also be mapped in quantitative trait loci (QTL) studies that provide evidence of genetic regulation of gene expression (eQTL) (11,12) or protein abundance (pQTL) (13). Secondly, integrating genetic findings with protein interaction networks increases the chance of recovering existing therapeutic targets (5,14), highlighting the usefulness of protein interactions during drug development.

Priority index or Pi, a genetics-led target prioritisation approach (5), has already supported specific applications for a wide range of diseases, including but not limited to: Alzheimer's disease (15), Dupuytren's disease (16), endometriosis (17), kidney stone disease (18), myasthenia gravis (19) and type 1 diabetes (20). The Pi approach is unique in three ways. Firstly, the approach is competitive compared with other genetics-based methods, according to performance benchmarking in recovering known drug targets for immune-mediated diseases (21). Secondly, the approach respects the omnigenic model for complex traits (22,23), considering target candidates that include core genes (directly linked to disease-associated variants using functional genomics) and peripheral genes (linked to core genes using knowledge of protein interactions). Lastly, the endpoint (and the uniqueness) of the Pi target prioritisation is the ability in identifying interconnected (or nodal) genes that mediate crosstalk between molecular pathways (24). Identification of pathway crosstalk is motivated by clinical interests in pathway-centric therapeutic targeting strategies, particularly targeting crosstalk genes (that is, meetpoints between closely related pathways).

In this study I present web-based facilities, namely ‘PiER’, allowing the users to perform genetic target prioritisation. The PiER is capable of *ab initio* genetic target prioritisation. The entire prioritisation process can be completed almost *real-time*, considering that a multi-step prioritisation process is typically required for complex tasks. This level of capacity from the PiER is not available elsewhere. Such capacity is lacking in the Priority index (21) and the Open Targets Genetics Portal (25), the most relevant to the PiER. Both allow the users to access pre-computed genetic targets stored in relational databases; in other words, integrative prioritisation using user-input data on the fly is not supported.

In the remaining sections, I first describe the PiER design, two facilities and implementation. I then detail the tasks supported in each of two facilities, with utilities illustrated using a real-world example [i.e. shared genetic variants identified from GWAS in inflammatory disorders (26)]. Finally, I discuss limitations of the PiER and the scope for future developments.

## MATERIALS AND METHODS

### Design

**Figure 1.**
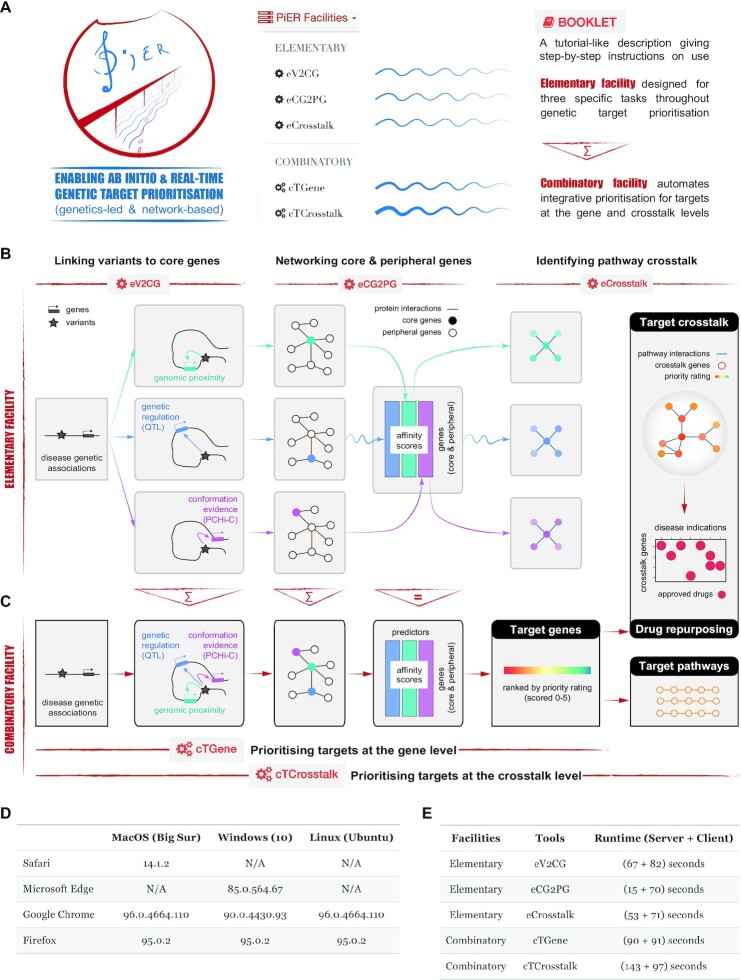
Design and facilities of the PiER. (**A**) The artwork of the same name is designed to resemble the PiER. The above-water pillar structure in red (symbolising the infrastructure) and water waves in blue (by analogy the piano stave consisting of five horizonal lines) collectively illustrate the web-based PiER facilities enabling *ab initio* and *real-time* genetic target prioritisation. Also illustrated is the tutorial-like booklet (in an HTML format) that describes step-by-step instructions on how to use. (**B**, **C**) Schematic illustration of five main tasks grouped into two facilities. The elementary facility performs specific tasks, including three online tools (*eV2CG*, *eCG2PG* and *eCrosstalk*), and each of them giving results is sequentially piped to the next one (B). By chaining together elementary tasks supported in the elementary facility, the combinatory facility performs complex tasks, including two online tools (*cTGene* and *cTCrosstalk*) (C). PCHi-C, promoter capture Hi-C; QTL, quantitative trait loci. (**D**) A summary of the PiER browser compatibility. N/A, not available; otherwise, the browser version is stated. (**E**) A summary of the runtime (on the server and client sides) per tool estimated using Google Chrome.

By design, the PiER is simplistic but efficient, featuring two web-based facilities: *elementary* and *combinatory* (Figure 1A). As per the piano stave, the PiER consists of five horizontal lines in blue, with thinner lines representing the elementary facility designed to perform specific tasks throughout the prioritisation process, and thicker lines signifying the combinatory facility designed to automate integrative target prioritisation at both the gene and crosstalk levels. For ease of use, a tutorial-like booklet describing step-by-step instructions in detail is provided where needed.

### Elementary and combinatory facilities

A schematic overview of two facilities supported in the PiER is illustrated in Figure 1B and C. The elementary facility supports specific tasks (Figure 1B), including three online tools: (i) *eV2CG*, utilising functional genomics to link disease-associated variants (including those located at the non-coding genome) to core genes likely responsible for genetic associations; (ii) *eCG2PG*, using knowledge of protein interactions to ‘network’ core genes with each other and with additional peripheral genes as well, producing a ranked list of core and peripheral genes and (iii) *eCrosstalk*, exploiting the information of pathway-derived interactions to identify highly-ranked genes that mediate crosstalk between molecular pathways. By chaining together elementary tasks supported in the elementary facility, the combinatory facility enables automation of genetics-led and network-based integrative prioritisation for genetic targets, both at the gene level (*cTGene*) and at the crosstalk level (*cTCrosstalk*) (Figure 1C). In addition to target crosstalk, the *cTCrosstalk* also supports target pathway prioritisation and crosstalk-based drug repurposing analysis (i.e. repositioning clinically approved drugs from original disease indications into new ones).

### Implementation

The PiER was developed using a next-generation Perl web framework ‘Mojolicious’ that requires nearly zero-effort maintenance for interface updates. The PiER was also built using ‘Bootstrap’ that supports the mobile-first and responsive webserver in all major platform browser**s** (Figure 1D). All tasks using online tools in the PiER can be completed within three minutes on the server side (Figure 1E), though the estimated runtime on the client side varies depending on the users’ broadband connection speed.

## RESULTS

### Elementary facility: *eV2CG* — linking variants to core genes

The task of the *eV2CG* is to link disease-associated variants (SNPs) to core genes likely responsible for genetic associations (Figure 1B, left panel). The input includes two pieces of information: dbSNP rsIDs (27) and the significance info (p-values). For example, 244 SNPs and their reported p-values for inflammatory disorders (26) are used as an illustrative example in the user-request interface. Input SNPs with a typical threshold (*P*-value < 5 × 10^−8^) are considered. Additional SNPs in linkage disequilibrium (*R*^2^ > 0.8) can be also included, by default, according to the European population. Other populations (i.e. African, Admixed American, East Asian, and South Asian) are also supported (28). These SNPs are then used to define core genes based on genomic proximity, e/pQTL or PCHi-C. Functional genomic datasets currently in support include blood eQTL from the eQTLGene Consortium (12), plasma pQTL (13) and PCHi-C in 17 primary blood cell types (29). As previously described (5), the scoring for core genes considers: (i) disease genetic associations (p-values, the threshold and *R*^2^ for SNPs); (ii) distance-to-SNP window for genomic proximity; (iii) the significance level of genetic association with gene expression for eQTL datasets (or protein abundance for pQTL datasets); and (iv) the strength of gene promoters physically interacting with SNP-harbouring genomic regions for PCHi-C datasets. The output includes a manhattan plot and a tabular display, both used to illustrate core genes and their scores (quantifying the level of core genes responsible for disease genetic associations). Also provided is an evidence table showing which SNPs are used to define core genes based on which datasets.

### Elementary facility: *eCG2PG* — networking core genes to peripheral genes

The *eCG2PG* is tasked to ‘network’ core genes with each other and with additional peripheral genes as well using knowledge of protein interactions (Figure 1B, middle panel). Protein interactions are sourced from the STRING database (version 11.5 in August 2021) only with source codes ‘experiments’ and ‘databases’ (30). The database has interaction score thresholds of increasing confidence (0.4 for the medium confidence, 0.7 for the high confidence, and 0.9 for the highest confidence). By default, the high-confidence interactions are considered, corresponding to a total of ∼14 000 nodes/genes and ∼202 000 interactions/edges used for the networking. With core genes used as seeds, the random walk with restart algorithm (31) was implemented to identify (non-seed) peripheral genes under network influence, leveraging protein interaction network information. A non-seed peripheral gene with higher connectivity to core genes (seeds) will receive a higher affinity score, and a highly networked core gene will receive a much higher affinity score. Taken together, the *eCG2PG* takes as inputs core genes (together with positive weights, such as core gene scores resulted from the *eV2CG*), and outputs a list of core and peripheral genes (ranked by affinity scores quantifying the network connectivity to input core genes).

### Elementary facility: *eCrosstalk* — identifying pathway crosstalk

The *eCrosstalk* is designed to exploit the information of well-curated pathway-derived interactions to identify pathway crosstalk from an input ranked list of genes (Figure 1B, right panel). Pathway-derived interactions are derived by merging pathways from the KEGG database (release 101.0 in January 2022) (32), collectively forming a gene network in which each interaction/edge is found in at least one pathway. The objective of the *eCrosstalk* is to identify a subset of the gene network in a way that the resulting subnetwork (or ‘pathway crosstalk’) contains highly-ranked genes. This task is achieved via heuristically solving a prize-collecting Steiner tree problem, the solver demonstrated to outperform other state-of-the-art algorithms (33,34). The significance of the identified crosstalk is estimated by a degree-preserving node permutation test (34). More importantly, the users can specify a desired number of nodes/genes in the resulting crosstalk, with the desired output returned via a well-established iterative search procedure (34). In summary, the input is a ranked list of genes (together with positive scores, such as affinity scores resulted from the *eCG2PG*), and the output includes a tabular display of crosstalk genes and a network-like visualisation of the crosstalk (with nodes/genes colour-coded by input scores).

### Combinatory facility: *cTGene* — prioritising targets at the gene level

**Figure 2.**
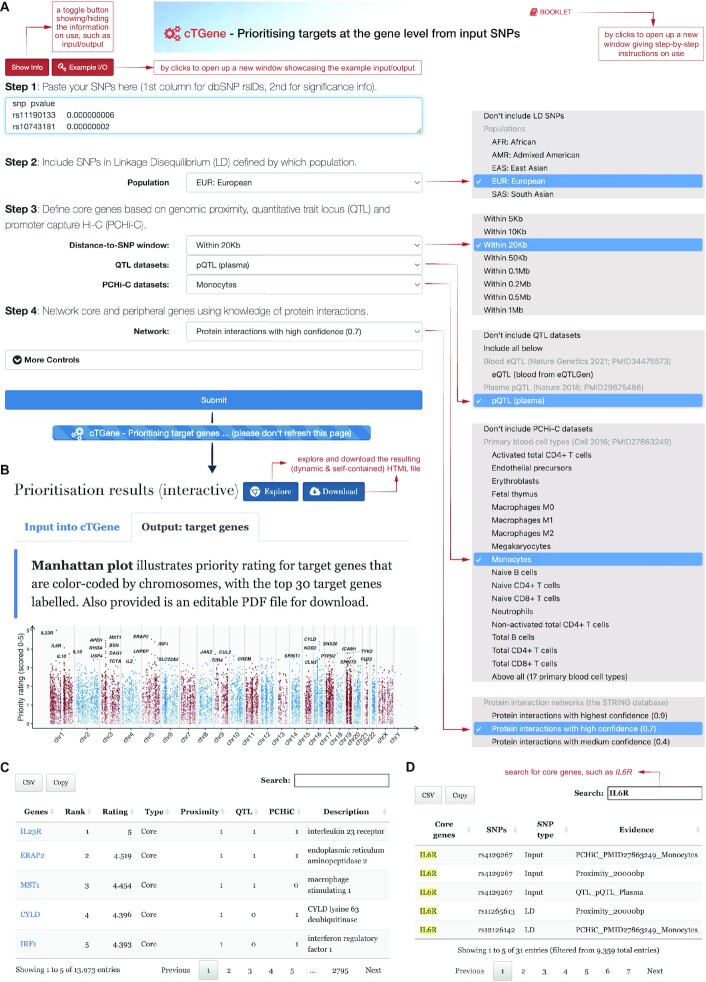
Prioritising target genes using the ‘*cTGene*’. (**A**) The user-request interface is designed in a manner that corresponds to the multi-step prioritisation process (left panel). Per specification, the available options are illustrated in the right panel. (B–D) The prioritisation results page. Under the tab ‘*Output: target genes*’ are a manhattan plot illustrating priority rating for target genes (**B**) and two tabular displays about prioritisation (**C**) and evidence (**D**). In the prioritisation table (**C**), the ‘Type’ column tells the target gene type (core genes *versus* peripheral genes), and the next three columns give a summary of evidence used to define core genes, including evidence of genomic proximity (non-zero number in the ‘Proximity’ column), e/pQTL evidence (the ‘QTL’ column) and conformation evidence (the ‘PCHiC’ column). In the evidence table (**D**), the ‘SNP type’ column is indicative of use-input SNPs *versus* LD SNPs, and the ‘Evidence’ column provides details on evidence (SNPs in the proximity, PCHi-C and e/pQTL datasets). LD, linkage disequilibrium.

The *cTGene* is specially tasked to automate genetics-led and network-based integrative prioritisation of target genes (Figures 1C and 2). Using real-world GWAS summary-level data from inflammatory disorders (26), I showcase this complex task to illustrate what the users need to provide and what can be expected. In corresponding to the multi-step prioritisation process, the user-request interface (Figure 2A) takes as inputs disease-associated SNPs and their significance level, and requires the users to specify the following information: (i) whether to include SNPs in linkage disequilibrium (and if included, based on which population); (ii) how to define core genes in terms of the evidence of genomic proximity, QTL and PCHi-C (i.e. conformation evidence); and (iii) which protein interactions used for ‘networking’ core and peripheral genes. The interface also allows the users to specify additional parameters for more controls over the prioritisation process and results.

The prioritisation results page provides a summary of input data and runtime (calculated on the server side), which can be found under the tab ‘*Input into cTGene*’. Under the tab ‘*Output: target genes*’, a manhattan plot is drawn to illustrate priority rating for ∼14 000 target genes across the genome (Figure 2B). Prioritised target genes are displayed in a table (Figure 2C), together with priority rank and rating (scored 0–5), the gene type (core genes *versus* peripheral genes), and a summary of evidence (proximity, QTL and PCHi-C). The evidence table shows which SNPs are used to define core genes based on which evidence (Figure 2D). For example, the user-input SNP ‘rs4129267’ is linked to the core gene *IL6R*, supported by multiple lines of evidence from genomic proximity, monocyte PCHi-C and plasma pQTL.

The users are referred to the Pi approach publication (5) and benchmarking results (21) for details on how to calculate, interpret and validate the priority rating. In brief, the priority rating is calculated in an unsupervised manner using a Fisher's combined method applied to the gene-predictor matrix that contains affinity scores (illustrated in Figure 1C). A target gene supported by multiple lines of evidence receives a higher priority rating (i.e. highly prioritised). It differs from Open Targets in two ways. Firstly, the Open Targets approach uses a weighted harmonic sum strategy to combine data-source-specific scores (35), including the locus-to-gene score from the Open Targets Genetics Portal (25). Secondly, the locus-to-gene score uses machine learning (i.e. in a supervised manner) to link disease-associated variants to causal genes by integrating fine-mapping results and functional genomic datasets (25). Thirdly, using knowledge of molecular interactions to identify functionally linked targets (i.e. peripheral genes without direct genetic evidence) is not supported in Open Targets (35).

It is highly recommended to consider the top 1% prioritised target genes for downstream analyses and interpretations, for example, used to further prioritise target pathways (see the next section). The validity of the priority rating has been empirically demonstrated by showing high correlations to experimentally measured target activities, with the significance estimated using a randomised test; see (5) for details. The validity has been further illustrated for immune-mediated diseases according to performance benchmarking (21), outperforming other genetics-based methods (including Open Targets) and Naïve prediction (i.e. prioritising a gene by how often the gene has been targeted by existing approved drugs).

In this showcase, the top prioritised target genes are essential for inflammation, and more interestingly, have been previously reported to be associated with inflammatory disorders, such as *IL23R* [ranked 1st; well-known as an inflammatory bowel disease gene (36)], *ERAP2* [2nd; associated with ankylosing spondylitis (37)] and *MST1* [3rd; associated with primary sclerosing cholangitis (38)]. All prioritised target genes are cross-referenced and linked to GeneCards (39). Notably, all prioritisation results are embedded into a dynamic and self-contained HTML file, which can be either interactively explored in a new browser window or downloaded for the exploration afterwards. The ‘Show/Hide Info’ toggle button contains the help information on use, including the details on input, output, mechanism and other useful information, while the ‘Example I/O’ button showcases the example input/output.

### Combinatory facility: *cTCrosstalk* — prioritising targets at the crosstalk level

**Figure 3.**
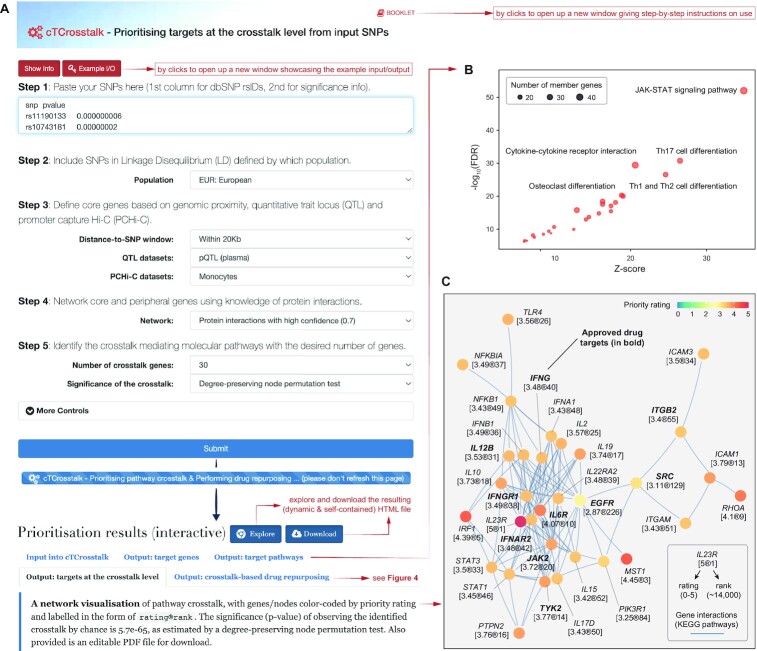
Prioritising targets at the crosstalk level using the ‘*cTCrosstalk*’. (**A**) The user-request interface and the prioritisation results page. In addition to a summary of input data and the runtime (computed on the server side) under the tab ‘*Input into cTCrosstalk*’, the prioritisation results page provides the output, including target genes (the same as in Figure 2B–D), target pathways, targets at the crosstalk level, and crosstalk-based drug repurposing (see Figure 4). (**B**) A dot plot for prioritised target pathways, with the top five labelled, available under the tab ‘*Output: target pathways*’. (**C**) A network visualisation of the crosstalk, with genes/nodes colour-coded by priority rating and labelled in the form of ‘rating®rank’, available under the tab ‘*Output: targets at the crosstalk level*’. Notably, also available are two tabular displays about prioritisation and evidence for crosstalk genes (not illustrated here as similarly shown in Figure 2C and D).

**Figure 4.**
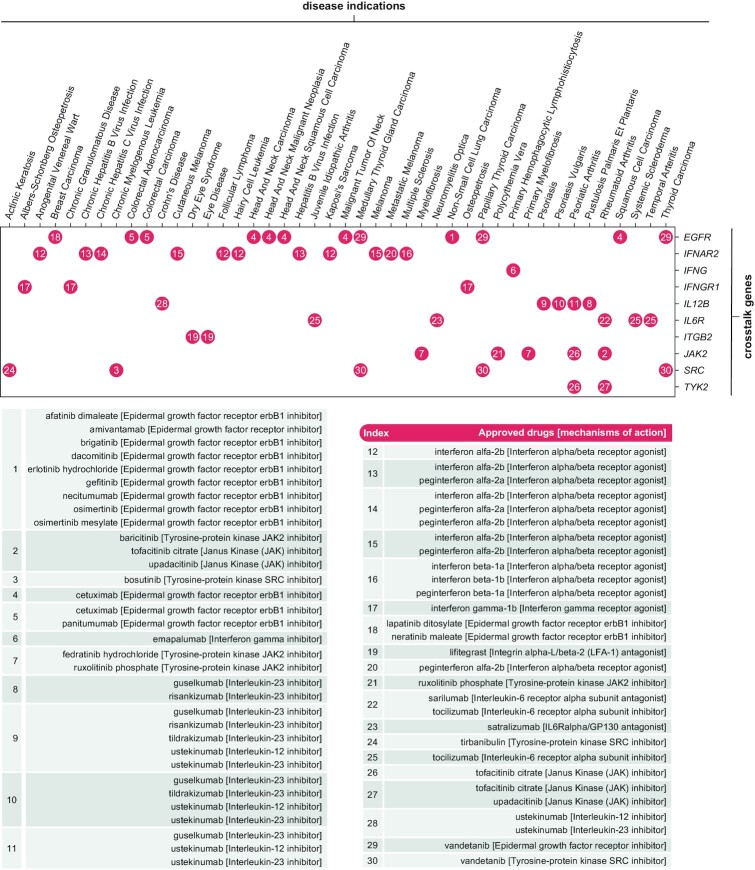
Crosstalk-based drug repurposing using the ‘*cTCrosstalk*’. Available under the tab ‘*Output: crosstalk-based drug repurposing*’ is a heatmap-like illustration, with crosstalk genes on the y-axis, disease indications on the x-axis, and red dots indexed in numbers. The index numbers are referenced in a table where the information on approved drugs and mechanisms of action is detailed.

As an extension to the *cTGene*, the *cTCrosstalk* continues to prioritise target pathways, identify crosstalk mediating molecular pathways, and perform crosstalk-based drug repurposing (Figures 1C, 3 and 4). The user-request interface is identical to the ‘*cTGene*’ (illustrated in Figure 2A), except for additional specifications that control the desired number of crosstalk genes and the significance of the identified crosstalk (Figure 3A). Using the same example described in the previous section, the *cTCrosstalk* not only prioritises target genes, but also outputs target pathways that are prioritised based on enrichment analysis of the top 1% (by default) prioritised target genes using KEGG (32). As illustrated in a dot plot (Figure 3B), the top prioritised pathway is the JAK-STAT signalling, which aligns with current interests targeting this pathway in inflammatory and autoimmune diseases (40), particularly for treating inflammatory bowel disease (41). The member genes of this top pathway can be retrieved under the tab ‘*Output: target pathways*’.

The underlying PiER approach is unique in its ability to identify a concise and manageable list of pathway crosstalk genes, the endpoint list of genetic target prioritisation [see the review (24)]. The list also provides opportunities for drug repurposing [see the review (42)]. Accordingly, under the tab ‘*Output: targets at the crosstalk level*’, the output crosstalk is visualised, with genes/nodes colour-coded by priority rating and labelled in the form of ‘rating®rank’ (Figure 3C). The significance (*P*-value) of observing the identified crosstalk by chance is 5.7 × 10^−65^, as estimated by a degree-preserving node permutation test (5). Interestingly, the crosstalk hub nodes (*EGFR*, *IFNAR2*, *IFNGR1*, *IL22RA2*, *IL23R*, *IL6R, JAK2* and *TYK2*) are all key players of the JAK-STAT signalling, further supporting the potential of therapeutic intervention targeting this pathway.

Under the tab ‘*Output: crosstalk-based drug repurposing*’, a heatmap-like illustration shows the output from drug repurposing analysis based on crosstalk genes (Figure 4). This showcase identifies 10 genes (*EGFR*, *IFNAR2*, *IFNG*, *IFNGR1*, *IL12B*, *IL6R*, *ITGB2*, *JAK2*, *SRC* and *TYK2*) of licensed medications (approved drugs already in clinical use). The information on current approved therapeutics is sourced from the ChEMBL database (version 30 in March 2022) (43), including drugs, disease indications, and non-promiscuous targets that explain the efficacy of drugs in disease with well-defined mechanisms of action. Together with the information on mechanisms of action detailed in an interactive table beneath, the users can explore drug candidates to seek repurposing opportunities.

## DISCUSSION

In a new era of human disease genetics research and drug development, the focus has been rapidly shifted towards translational use of genetic findings to reduce drug attrition rate along the drug discovery pipeline. Integrative prioritisation for genetic targets is the key to this shift, as highlighted by an early successful example (14). Genetic evidence arising from human disease genomics can inform drug target discovery, for which web-based implementation is much needed (which is also challenging). The PiER, specially designed for genetic target prioritisation and implemented *ab initio* and *real-time*, contributes significantly to accelerating early-stage target discovery and drug repurposing. In addition to target prioritisation at the gene level, target identification at the crosstalk level provides the users with actionable numbers of target candidates and clinically approved drugs that can be taken forward for exploring drug repurposing opportunities.

The PiER is largely limited by available functional genomic datasets that are the key in linking non-coding variants to core genes responsible for genetic associations. Since functional genomic datasets in support are mostly immune-related, the PiER is particularly useful to prioritise genetic targets for diseases with the immune component. Precaution should be taken when applying to disease areas where e/pQTL and PCHi-C datasets are not directly relevant. Accordingly, my first aim in future developments is to expand the supporting data required for the PiER; this includes functional genomic datasets involving a diverse range of cell types (11,44), particularly expanding to the data for non-immune disorders. The second aim is to incorporate target tractability, another important component for target discovery that is not currently supported by the PiER. Tractability is to assess the possibility of being targeted by small molecules, antibodies, or proteolysis-targeting chimeras (35,45). In the long term, the PiER serves as an interactive platform that promotes collaborative efforts to rapidly advance computational translational medicine that leverages human disease genetics and genomics for target discovery and drug repurposing.

## DATA AVAILABILITY

The PiER can be accessed at http://www.genetictargets.com/PiER, with the booklet made available at http://www.genetictargets.com/PiER/booklet. The source codes behind the PiER are deposited into GitHub (https://github.com/23verse/pier).
